# Percutaneous Closure of Side-to-Side Femoral Arteriovenous Fistula Using Untied Suture

**DOI:** 10.1016/j.jaccas.2025.103955

**Published:** 2025-07-09

**Authors:** Nour W. Alomari, Ahmad I. Alomari, Mohammad Amarneh

**Affiliations:** a University of Nebraska, Omaha, Nebraska, USA; b Division of Vascular and Interventional Radiology, Boston Children’s Hospital, Boston, Massachusetts, USA; c Harvard Medical School, Boston, Massachusetts, USA

**Keywords:** congenital heart defect, iatrogenic femoral arteriovenous fistula, minimally invasive closure

## Abstract

Side-to-side femoral arteriovenous fistulas are not amenable to closure with embolization, and surgical repair remains the traditional treatment. A 16-month-old boy presented with swelling in his right lower extremity, a complication arising from a side-to-side arteriovenous fistula that developed after cardiac catheterization at 5 months of age. The iatrogenic fistula was effectively closed using a simple technique involving disruption of the fistula with a curved needle and the placement of a transfistula untied suture under sonographic guidance. A follow-up visit 4 years later indicated the fistula remained obliterated.

## Graphical Abstract

**Figure:**
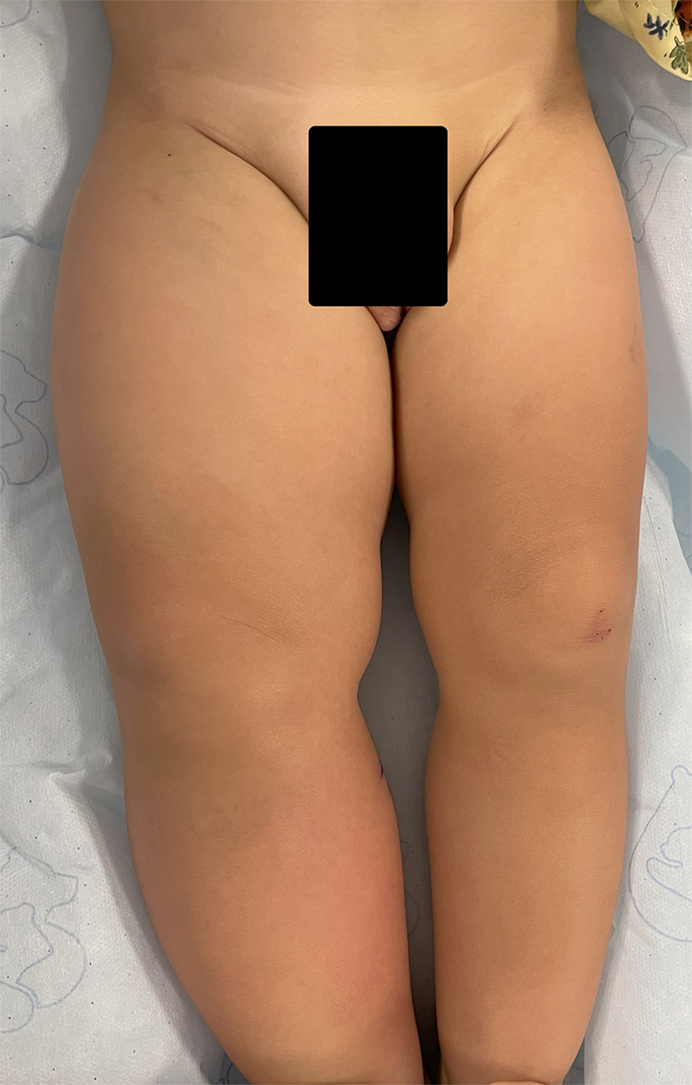

The clinical sequelae of undiagnosed arteriovenous fistulas (AVFs) are significant and include dilatation of the vessels, venolymphatic trophic complications, and heart failure. Surgical vascular repair in children can be more challenging due to the small diameter of the injured vessel, the spasticity of young arteries, and the inherent difficulties of choosing a treatment that should be adapted to the continuous axial growth.^1^ Persistent and symptomatic iatrogenic AVFs can be treated with surgical repair or minimally invasive methods such as ultrasound-guided compression, stent placement, and embolization.^2^

**Take-Home Messages**

• Undiagnosed femoral iatrogenic arteriovenous fistulas in children have specific clinical features.
• An alternative percutaneous intervention for closure of side-to-side arteriovenous fistulas uses simple tools.

## Case Summary

**Figure 1:**
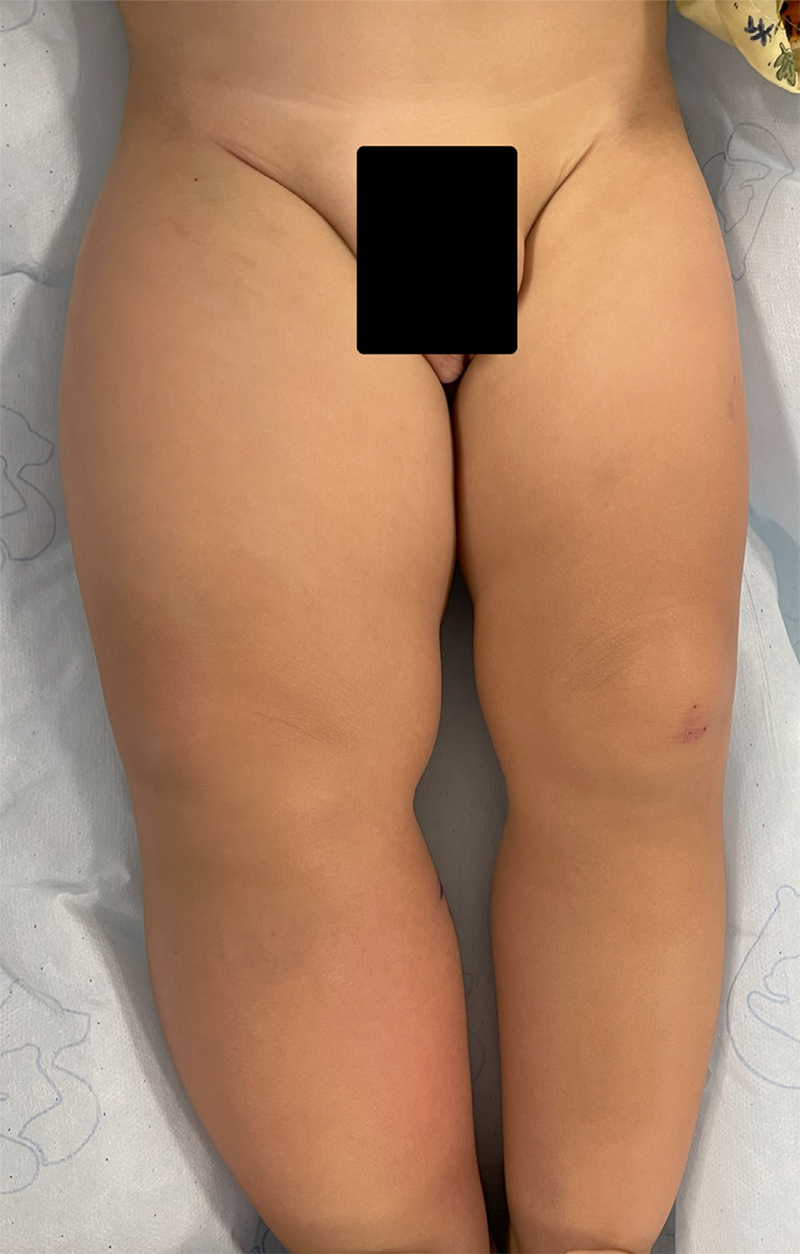
Diffuse Enlargement of the Right Lower Extremity

A 16-month-old boy with hypoplastic left heart syndrome was referred to our center for evaluation of swelling of the right lower extremity. He had undergone 2 cardiac catheterizations at 4 and 5 months of age with access via the left common femoral artery and right common femoral vein. After the last procedure, he developed gradual enlargement of the right lower extremity with erythematous purplish discoloration with standing (Figure 1), which was initially attributed to potential lymphedema. Examination showed generalized swelling of the entire lower extremity with a 5-cm discrepancy in thigh circumference, dilated superficial veins, and a thrill in the right groin.

The child had been born with mitral stenosis and aortic atresia variant of hypoplastic left heart syndrome, partial anomalous pulmonary venous connection (PAPVC) of the right upper pulmonary vein to the right superior vena cava, and aberrant origin of the left circumflex coronary artery from the right pulmonary artery. He underwent stage I palliation, bidirectional Glenn shunt procedure, transfer of anomalous circumflex coronary artery from the right pulmonary artery to the aortic root, patch augmentation of the aortic root, complex patch reconstruction of the pulmonary artery as well as patch reconstruction of the superior vena cava, PAPVC, and tricuspid valve at the age of 7 months.

The differential diagnosis of unilateral lower limb enlargement in a child includes venous hypertension, lymphedema, vascular anomalies (venous malformation, lymphatic malformation, arteriovenous shunts) and overgrowth syndromes such as Klippel-Trenaunay syndrome, CLOVES (congenital lipomatous overgrowth vascular malformations, epidermal nevi, spinal/skeletal anomalies), Parkes-Weber syndrome, and hemihypertrophy.

**Figure 2:**
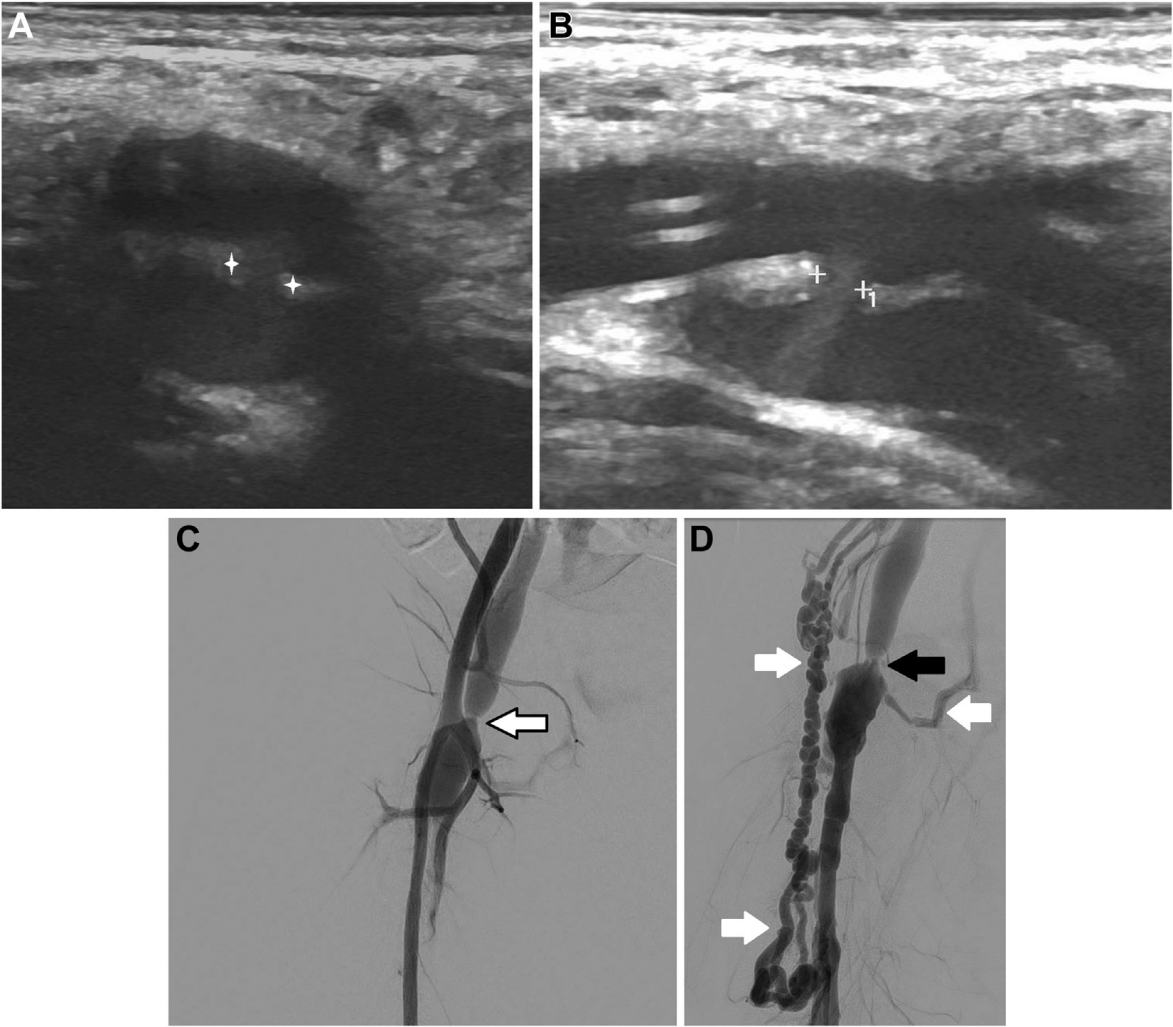
Imaging Findings (A) Transverse and (B) longitudinal grayscale ultrasound images demonstrating the side-to-side fistula between the dilated common femoral artery and posteriorly located vein measuring 1.6 mm. (C) Right iliac angiography: immediate shunting from the dilated right common femoral artery into an aneurysmal portion of the common femoral vein with proximal venous stenosis (arrow). (D) Ascending venography demonstrating severe stenosis of the right external iliac vein (black arrow) and collateral venous drainage in downward and upward directions (white arrows).

Ultrasonography showed a direct side-to-side fistula between the posterior wall of the dilated common femoral artery and the anterior wall of the vein with no discernable space between the vessels (Figures 2A and 2B). Angiography and closure of the AV fistula were planned under general anesthesia. Access to the left common femoral artery was made under sonographic guidance, and a 3.3-F JL Mongoose catheter (Pediavascular) was advanced into the right common iliac artery over a 0.025-inch (0.064-cm) angled hydrophilic guidewire (Radiofocus Wire; Terumo). Angiography demonstrated the side-to-side fistula between the dilated common femoral artery and stenotic common femoral vein (Figure 2C). Given the suboptimal venous opacification, venography of the lower extremity was performed, demonstrating direct shunting into an aneurysmal dilated segment of the femoral vein measuring 3 × 1.5 cm with severe stenosis of the external iliac vein (Figure 2D). The stenosis was likely caused by the venous access during the catheterization procedures. Large antegrade and retrograde collateral veins were noted in the thigh, pelvis, and abdominal wall.

**Figure 3:**
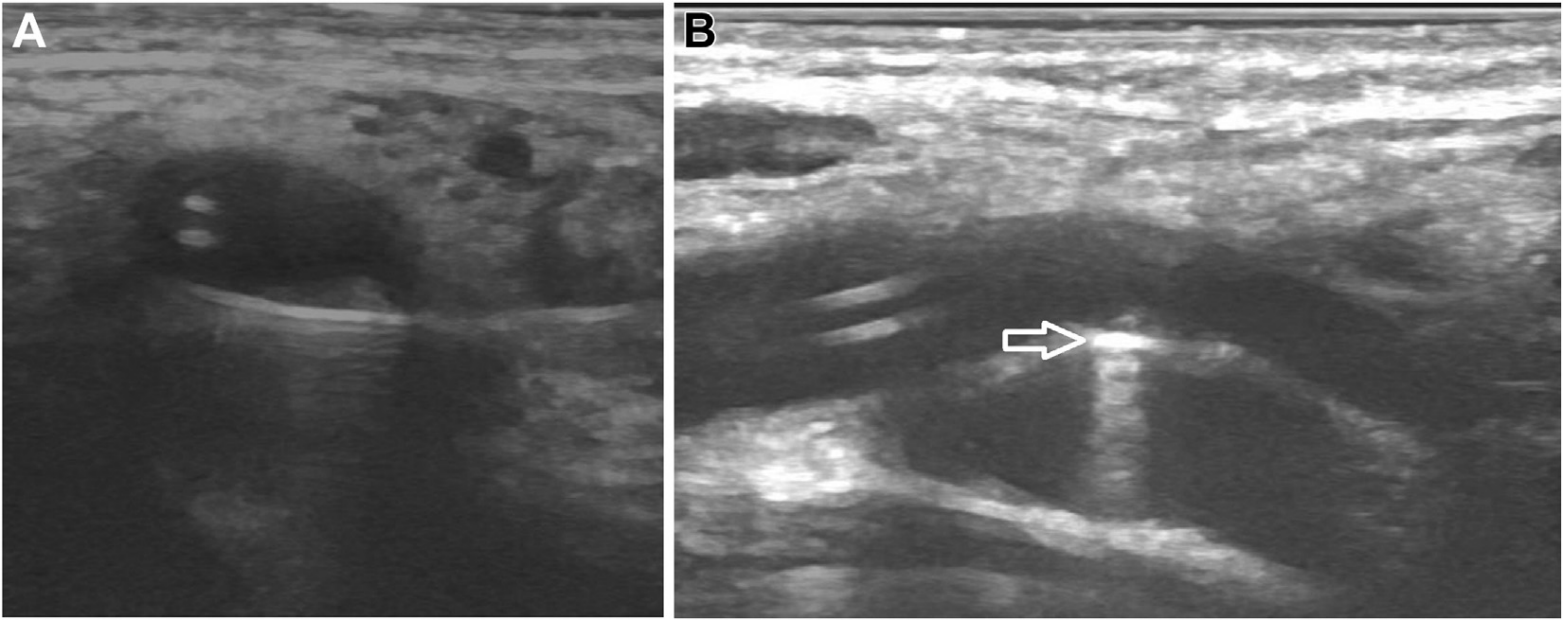
Sonographic-Guided Needle Placement (A) Transverse and (B) longitudinal ultrasound images demonstrating needle placement through the fistula (arrow).

**Figure 4:**
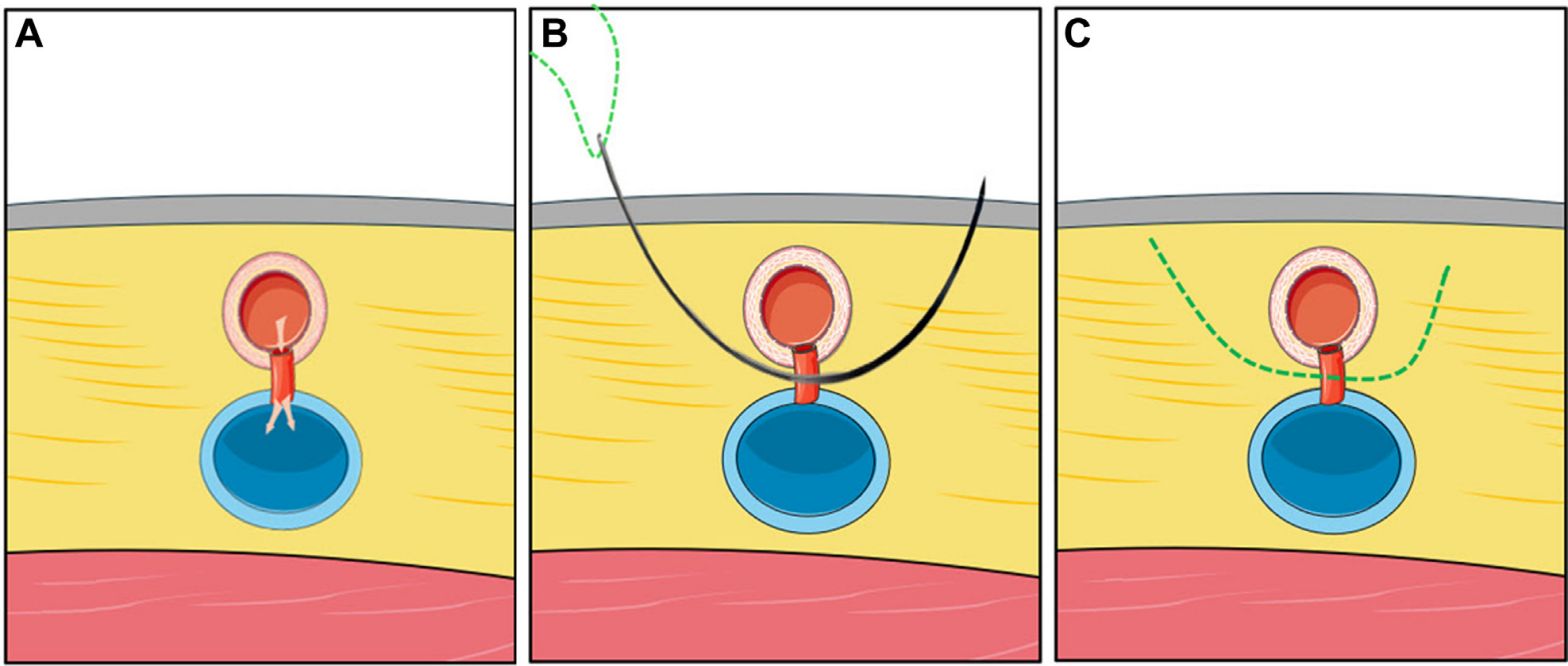
Axial Cross-Sectional Illustration Depicting the Closure of the Arteriovenous Fistula Using an Untied Suture Technique (A) The short arteriovenous fistula between the femoral artery (red) anteriorly and vein (blue). (B) A long, curved needle with an absorbable suture is percutaneously introduced from one side and precisely aligned to traverse the fistula between the artery and the vein and exit on the contralateral side. (C) The suture is subsequently divided on both ends and remains untied within the fistula.

Because of the lack of adequate tract length for safe embolization, the decision was made to disrupt the fistula. A slightly curved 7-cm, 21-gauge EchoTip needle (Cook Medical) was inserted via the skin lateral to the level of the fistula. The needle was directed toward the narrow space between the artery and the vein under sonographic guidance. The needle was carefully aligned to traverse the fistula and exit the skin medially while avoiding the femoral vessels (Figure 3). The needle was exchanged over 0.018-inch (0.046-cm) nitinol wire for a 3-F vascular introducer (Cook Medical). The sonographic examination at this point demonstrated no flow through the AVF. To ensure persistent closure of the fistula, a doubled 2-0 polyglactin 910 absorbable suture (Vicryl; Ethicon) was inserted through the introducer. Notably, this technique could have been performed practically using a suture-fitted, curved, long, free-eyed needle such as the curved King needle or straight cutting point Keith needle with an added curve (Aspen Surgical) (Figure 4).

**Figure 5:**
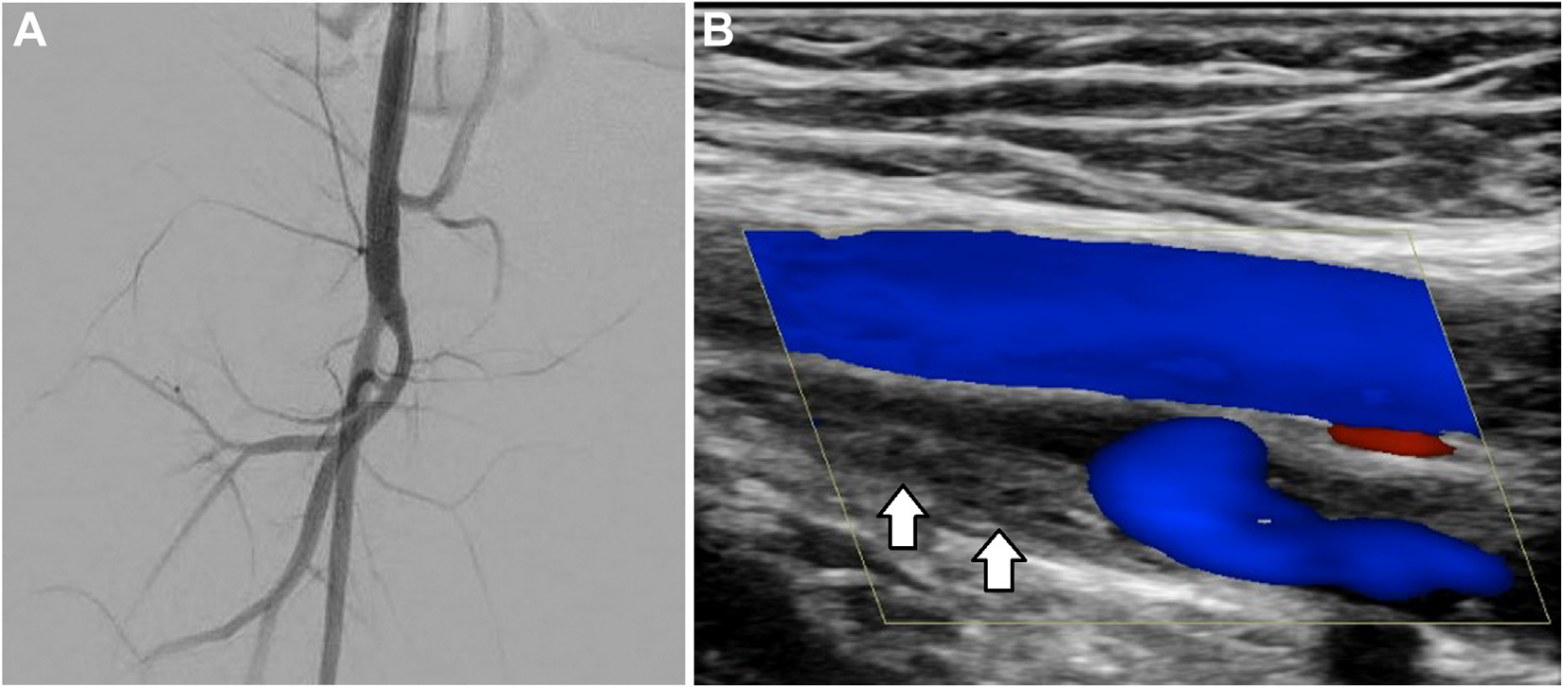
Post-Procedural Outcome (A) Angiography immediately after the needle interruption and placement of untied suture revealing complete sealing of the arteriovenous fistula. (B) Longitudinal ultrasound image 4 years and 2 months after treatment: patent femoral artery and partially occlusive chronic thrombus in the femoral vein (arrows).

After removal of the introducer, the external ends of the suture were cut. Complete closure of the arteriovenous fistula was confirmed with ultrasonography and angiography (Figure 5A).

## Discussion

Though surgical repair remains the traditional treatment of vascular trauma in children, both surgical and interventional methods are challenged by the small size of the injured vessels, the risk of spasm, and the inherent challenge of the normal growth of vessels.^1^^,^^3^ Although iatrogenic femoral AVFs with short tracts can be closed using balloon-assisted embolization,^3^ side-to-side fistulas with no tract are not amenable to such intervention. Placement of an arterial covered stent to obliterate an AVF is technically feasible for side-to-side fistulas,^4^ but this is not optimal for children with small vessels, and it requires a large arterial access.

To the best of our knowledge, this is the first reported experience of minimally invasive closure of iatrogenic side-to-side AVF using sonographic-guided disruption of the AVF tract. We believe that this novel technique may offer a simple, safe, and cost-effective alternative to surgical repair, with immediate results and fewer complications. The procedure is short and requires widely available, inexpensive supplies. Furthermore, it can potentially be performed without catheter angiography or general anesthesia in cooperative patients.

## Follow-up

Two months after the procedure, the thigh circumference discrepancy had decreased from 5 cm to 4 cm with no discoloration of the limb or thrill. Ultrasonographic examination demonstrated the occlusion of the fistula, a patent femoral artery, and a partially occlusive organized thrombus in the dilated segment of the femoral vein. Ultrasonography confirmed occlusion of the fistula, patency of the femoral artery, and a partially occlusive thrombus in the dilated femoral vein segment. These findings persisted at a follow-up of 4 years and 2 months (Figure 5B).

## Conclusions

Closure of a femoral side-to-side femoral arteriovenous fistula using needle disruption and an untied transfistula suture is a novel technique, and this appears to be the first published experience. This minimally invasive method is a technically simpler, safer, and faster alternative to surgical repair.

## Funding Support and Author Disclosures

The authors have reported that they have no relationships relevant to the contents of this paper to disclose.

## References

[1] Lozano-Corona R., Torres-Machorro A., Ortiz-Beitz R. (2023). Review of surgical treatment of iatrogenic iliofemoral artery injury in the pediatric population after catheterization. Eur J Med Res.

[2] Kelm M., Perings S.M., Jax T. (2002). Incidence and clinical outcome of iatrogenic femoral arteriovenous fistulas: implications for risk stratification and treatment. J Am Coll Cardiol.

[3] Wang W., Moon E., Spain J. (2013). Balloon-assisted N-butyl-2-cyanoacrylate closure of an iatrogenic femoral arteriovenous fistula. Vasc Endovascular Surg.

[4] Rama-Merchan J.C., Cruz-González I., Martín-Moreiras J. (2017). Percutaneous closure of iatrogenic femoral arteriovenous fistula using a covered coronary stent. Rev Port Cardiol.

